# Norfloxacinium nitrate

**DOI:** 10.1107/S2414314624008137

**Published:** 2024-08-30

**Authors:** Abdusamat Rasulov, Batirbay Torambetov, Bekmurod Alimnazarov, Shakhnoza Kadirova, Jabbor Suyunov, Yusufjon Nazarov, Jamshid Ashurov

**Affiliations:** a Institute of Bioorganic Chemistry, Academy of Sciences of Uzbekistan, M. Ulugbek St 83, Tashkent, 100125, Uzbekistan; b National University of Uzbekistan named after Mirzo Ulugbek 4 University St Tashkent 100174 Uzbekistan; c Termez State University, Barkamol Avlod St 43, Termez, 190111, Uzbekistan; University of Aberdeen, United Kingdom

**Keywords:** crystal structure, norfloxacin, mol­ecular structure, hydrogen bonds

## Abstract

In the title salt [systematic name: 4-(3-carb­oxy-1-ethyl-6-fluoro-4-oxo-1,4-di­hydro­quin­olin-7-yl)piperazin-1-ium nitrate], C_16_H_19_FN_3_O_3_^+^·NO_3_^−^, proton transfer from nitric acid to the N atom of the piperazine ring of norfloxacin has occurred to form a mol­ecular salt. In the extended structure, N—H⋯O hydrogen bonds link alternating cations and anions into [100] chains, which are reinforced by aromatic π–π stacking inter­actions between the quinoline moieties of the norfloxacinium cations.

## Abstract 2

The components of the title mol­ecular salt are linked by N—H⋯O hydrogen bonds and aromatic π–π stacking inter­actions.

## Graphical abstract

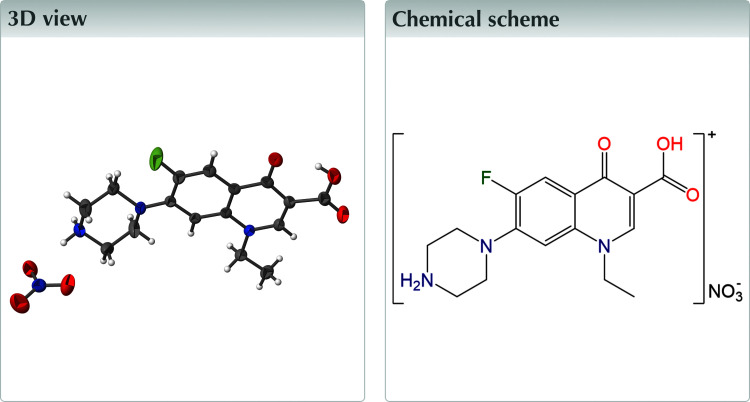

## Structure description

Norfloxacin (NF, C_16_H_18_N_3_O_3_F) is a synthetic fluoro­quinolone anti­biotic that has been used to treat a wide variety of bacterial infections since its introduction in the 1980s. It is effective against both Gram-positive and Gram-negative bacteria, and it has been shown to be particularly useful in the treatment of urinary tract infections, respiratory tract infections, and skin and soft tissue infections. Norfloxacin works by inhibiting the bacterial enzyme DNA gyrase, which is essential for DNA replication and transcription (Goldstein *et al.*, 1987 ▸; Mazuel, 1991 ▸; Chongcharoen *et al.*, 2008 ▸; Marc *et al.*, 2019 ▸; Spencer *et al.*, 2023 ▸). As part of our studies in this area we now describe the synthesis and structure of the title mol­ecular salt, C_16_H_19_N_3_O_3_F^+^·NO_3_^−^, (I), arising from the reaction of norfloxacin and nitric acid in aqueous solution.

**Figure 1:**
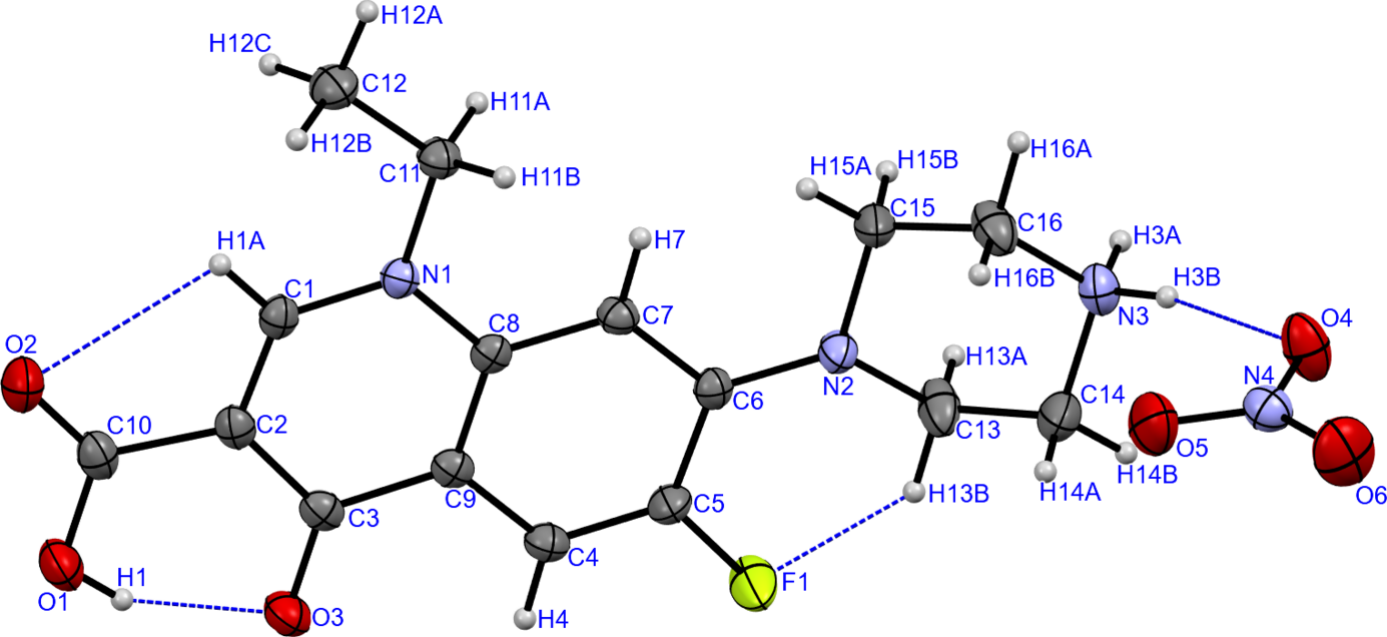
The mol­ecular structure of (I). Displacement ellipsoids are shown at the 50% probability level and hydrogen bonds are indicated by dashed lines.

Compound (I) crystallizes in the monoclinic space group *P*2_1_/*n*, with one cation and one anion in the asymmetric unit (Fig. 1 ▸). The N3 nitro­gen atom of the piperazine ring is observed to be protonated. In neutral NF, this nitro­gen atom is protonated by a hydrogen atom from the carb­oxy­lic acid moiety, resulting in a zwitterionic species (*e.g*., Gunnam & Nangia, 2023 ▸). However, in the crystal structure of (I), the hydrogen atom remains attached to the carb­oxy­lic acid fragment. This is evident from the significant difference (0.117 Å) in the lengths of the C10—O1 and C10—O2 bonds [1.325 (2) and 1.208 (2) Å, respectively]. In a delocalized carb­oxy­lic acid moiety, the C—O bond lengths are typically very similar, with a difference of only 0.006 Å (Razzoqova *et al.*, 2022 ▸). The atoms of the carboxyl moiety (C10, O1, and O2) and the quinoline moiety lie essentially in a plane, with maximum deviations from the mean plane of 0.029 (2) Å for O2 and 0.030 (2) Å for O1. The dihedral angle between the carboxyl and quinoline planes is 1.90 (19)°. The nitro­gen atom (N2) attached to the quinoline moiety is close to planar, as evidenced by the sum of bond angles around it being 356.5°. In contrast, the protonated nitro­gen atom (N3) adopts a tetra­hedral geometry. The piperazine ring exhibits a chair conformation. The ethyl substituent attached to N1 lies essentially in the plane of the quinoline moiety, as indicated by the C1—N1—C11—C12 torsion angle of 0.7 (2)°. The C5—F1 bond length of 1.3506 (18) Å is in good agreement with the mean value reported for 128 structures containing the NF moiety [*e.g*., 1.350 (2) Å reported by Sultana *et al.*, 2023 ▸]. Atom F1 accepts an intra­molecular hydrogen bond from H13*B* (C13—H13*B*⋯F1; Table 1 ▸), forming an *S*(6) ring. Another intra­molecular hydrogen bond is observed between H1 and O3 (O1—H1⋯O3), also forming a six-membered ring. Additionally, a weak hydrogen bond is present between H1*A* and O2 (C1—H1*A*⋯O2), forming a five-membered ring.

**Figure 2:**
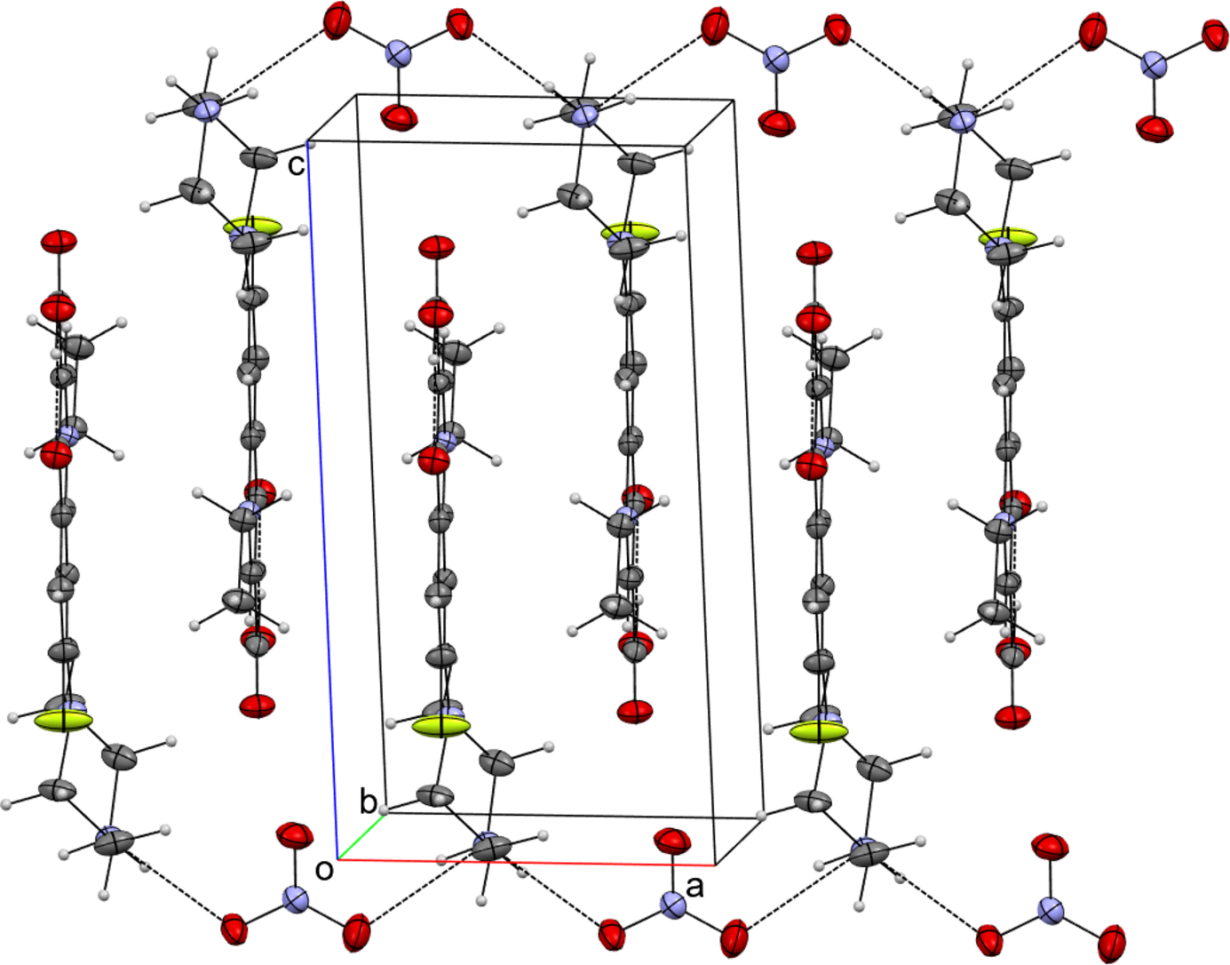
A fragment of a [100] chain in the extended structure of (I), with hydrogen bonds shown as dashed lines.

**Figure 3:**
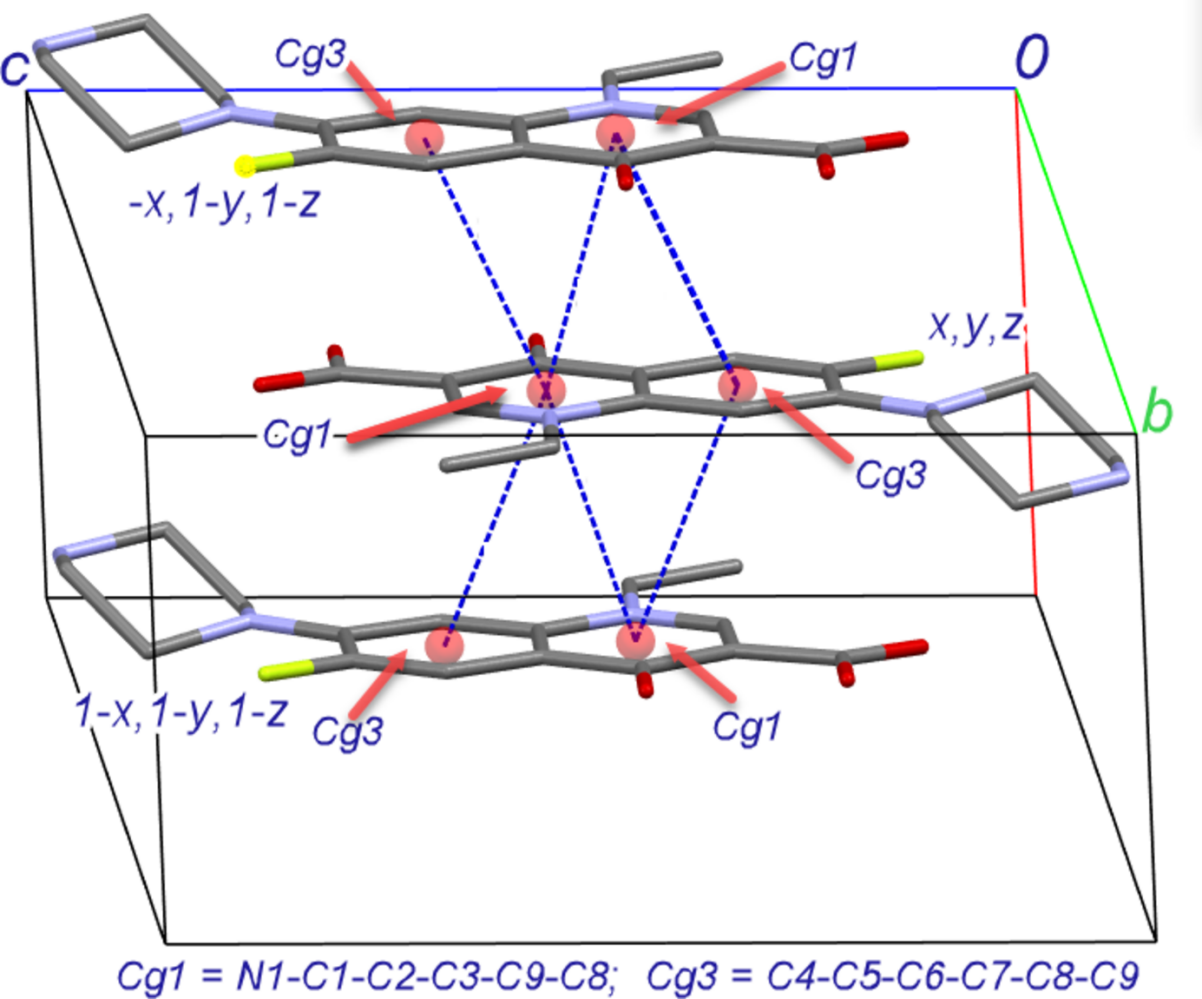
A view of the π–π stacking inter­action along the *a*-axis direction.

In the extended structure of (I), the norflaxacinium cation forms a hydrogen bond with the nitrate anion *via* its NH group (N3—H3*B*⋯O4). The nitrate anion, in turn, accepts a hydrogen bond from the NH group (N3—H3*A*⋯O6) of an adjacent NF cation related by the symmetry operation 1 + *x*, *y*, *z* (Table 1 ▸). These hydrogen bonds generate an infinite chain of alternating cations and anions propagating along the [100] direction (Fig. 2 ▸). This packing arrangement is repeated on the opposite side of the chain. As a result, strong π–π stacking inter­actions are formed between layers of NF cations facing each other (Fig. 3 ▸). The π–π stacking inter­actions are observed between the original NF cation and its symmetry-related counterparts located at −*x*, 1 − *y*, 1 − *z* and 1 − *x*, 1 − *y*, 1 − *z*. These inter­actions are highlighted by the short centroid–centroid distances: *Cg*1–*Cg*3(−*x*, 1 − *y*, 1 - *z)* is 3.6182 (8) Å and *Cg*1–*Cg*1 is 3.4403 (7) Å, and *Cg*1–*Cg*3(1 − *x*, 1 − *y*, 1 − *z)* is 3.5919 (8) Å and *Cg*1–*Cg*1 is 3.4862 (7) Å. These distances are notably shorter than the centroid–centroid contacts reported by Ibukun *et al.* (2023 ▸) and Shaikh *et al.* (2024 ▸). The angle between the mean planes of the quinoline moieties is zero by symmetry. These stacking inter­actions also contribute to the packing of mol­ecules along the [100] direction.

## Synthesis and crystallization

31.9 mg (0.1 mmol) of NF was dissolved in 5 ml of a 0.02 *M* nitric acid solution. The resulting clear solution was stirred at room temperature for 30 minutes. The solution was then transferred to a vial with small holes in the lid to allow for evaporation. After about a week, needle-like single crystals of the title salt suitable for data collection were obtained.

## Refinement

Crystal data, data collection and structure refinement details are summarized in Table 2 ▸.

## Supplementary Material

Crystal structure: contains datablock(s) I. DOI: 10.1107/S2414314624008137/hb4480sup1.cif

Structure factors: contains datablock(s) I. DOI: 10.1107/S2414314624008137/hb4480Isup2.hkl

Supporting information file. DOI: 10.1107/S2414314624008137/hb4480Isup3.cml

CCDC reference: 2378009

Additional supporting information:  crystallographic information; 3D view; checkCIF report

## Figures and Tables

**Table 1:** Hydrogen-bond geometry (Å, °)

*D*—H⋯*A*	*D*—H	H⋯*A*	*D*⋯*A*	*D*—H⋯*A*
O1—H1⋯O3	0.82	1.80	2.5640 (17)	154
N3—H3*B*⋯O4	0.89	1.97	2.8526 (19)	169
N3—H3*A*⋯O6^i^	0.89	2.08	2.937 (2)	161
C13—H13*B*⋯F1	0.97	1.96	2.726 (2)	135
C15—H15*A*⋯O4^ii^	0.97	2.47	3.252 (2)	138

Symmetry codes: (i) 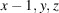; (ii) 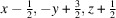.

**Table 2:** Experimental details

Crystal data	
Chemical formula	C_16_H_19_FN_3_O_3_^+^·NO_3_^−^
*M*_r_	382.35
Crystal system, space group	Monoclinic, *P*2_1_/*n*
Temperature (K)	293
*a*, *b*, *c* (Å)	6.6241 (1), 19.1629 (3), 12.6062 (2)
β (°)	93.136 (1)
*V* (Å^3^)	1597.80 (4)
*Z*	4
Radiation type	Cu *K*α
μ (mm^−1^)	1.12
Crystal size (mm)	0.12 × 0.06 × 0.06

Data collection	
Diffractometer	XtaLAB Synergy, Single source at home/near, HyPix3000
Absorption correction	Multi-scan (*CrysAlis PRO*; Rigaku OD, 2020 ▸)
*T*_min_, *T*_max_	0.977, 1.000
No. of measured, independent and observed [*I* > 2σ(*I*)] reflections	15217, 3087, 2593
*R*_int_	0.030
(sin θ/λ)_max_ (Å^−1^)	0.615

Refinement	
*R*[*F*^2^ > 2σ(*F*^2^)], *wR*(*F*^2^), *S*	0.046, 0.126, 1.05
No. of reflections	3087
No. of parameters	248
H-atom treatment	H-atom parameters constrained
Δρ_max_, Δρ_min_ (e Å^−3^)	0.30, −0.40

Computer programs: *CrysAlis PRO* (Rigaku OD, 2020 ▸), *SHELXT2019/3* (Sheldrick, 2015*a* ▸), *SHELXL2018/2* (Sheldrick, 2015*b* ▸) and *OLEX2* (Dolomanov *et al.*, 2009 ▸).

## full crystallographic data

### 4-(3-Carboxy-1-ethyl-6-fluoro-4-oxo-1,4-dihydroquinolin-7-yl)piperazin-1-ium nitrate Crystal data

**Table:**

C_16_H_19_FN_3_O_3_^+^·NO_3_^−^	*F*(000) = 800
*M**_r_* = 382.35	*D*_x_ = 1.589 Mg m^−^^3^
Monoclinic, *P*2_1_/*n*	Cu *K*α radiation, λ = 1.54184 Å
*a* = 6.6241 (1) Å	Cell parameters from 6698 reflections
*b* = 19.1629 (3) Å	θ = 4.2–71.2°
*c* = 12.6062 (2) Å	µ = 1.12 mm^−^^1^
β = 93.136 (1)°	*T* = 293 K
*V* = 1597.80 (4) Å^3^	Block, colourless
*Z* = 4	0.12 × 0.06 × 0.06 mm

### 4-(3-Carboxy-1-ethyl-6-fluoro-4-oxo-1,4-dihydroquinolin-7-yl)piperazin-1-ium nitrate Data collection

**Table:**

XtaLAB Synergy, Single source at home/near, HyPix3000 diffractometer	3087 independent reflections
Radiation source: micro-focus sealed X-ray tube, PhotonJet (Cu) X-ray Source	2593 reflections with *I* > 2σ(*I*)
Mirror monochromator	*R*_int_ = 0.030
Detector resolution: 10.0000 pixels mm^-1^	θ_max_ = 71.4°, θ_min_ = 4.2°
ω scans	*h* = −8→8
Absorption correction: multi-scan (CrysAlisPro; Rigaku OD, 2020)	*k* = −23→22
*T*_min_ = 0.977, *T*_max_ = 1.000	*l* = −15→15
15217 measured reflections	

### 4-(3-Carboxy-1-ethyl-6-fluoro-4-oxo-1,4-dihydroquinolin-7-yl)piperazin-1-ium nitrate Refinement

**Table:**

Refinement on *F*^2^	Primary atom site location: dual
Least-squares matrix: full	Hydrogen site location: inferred from neighbouring sites
*R*[*F*^2^ > 2σ(*F*^2^)] = 0.046	H-atom parameters constrained
*wR*(*F*^2^) = 0.126	*w* = 1/[σ^2^(*F*_o_^2^) + (0.0705*P*)^2^ + 0.357*P*] where *P* = (*F*_o_^2^ + 2*F*_c_^2^)/3
*S* = 1.05	(Δ/σ)_max_ = 0.001
3087 reflections	Δρ_max_ = 0.30 e Å^−^^3^
248 parameters	Δρ_min_ = −0.40 e Å^−^^3^
0 restraints	

### 4-(3-Carboxy-1-ethyl-6-fluoro-4-oxo-1,4-dihydroquinolin-7-yl)piperazin-1-ium nitrate Special details

**Table:**

Geometry. All esds (except the esd in the dihedral angle between two l.s. planes) are estimated using the full covariance matrix. The cell esds are taken into account individually in the estimation of esds in distances, angles and torsion angles; correlations between esds in cell parameters are only used when they are defined by crystal symmetry. An approximate (isotropic) treatment of cell esds is used for estimating esds involving l.s. planes.

### 4-(3-Carboxy-1-ethyl-6-fluoro-4-oxo-1,4-dihydroquinolin-7-yl)piperazin-1-ium nitrate Fractional atomic coordinates and isotropic or equivalent isotropic displacement parameters (Å^2^)

**Table:**

	*x*	*y*	*z*	*U*_iso_*/*U*_eq_	
F1	0.2303 (3)	0.44122 (6)	0.15882 (8)	0.0807 (5)	
O1	0.2724 (2)	0.35903 (6)	0.73719 (10)	0.0472 (4)	
H1	0.263362	0.345934	0.675190	0.071*	
O2	0.2684 (2)	0.45919 (7)	0.82376 (9)	0.0495 (4)	
O3	0.25105 (18)	0.35942 (6)	0.53350 (9)	0.0402 (3)	
N1	0.25398 (17)	0.57393 (6)	0.54595 (9)	0.0268 (3)	
N2	0.2332 (2)	0.59041 (7)	0.16174 (9)	0.0329 (3)	
N3	0.3057 (2)	0.67660 (7)	−0.01891 (11)	0.0377 (4)	
H3A	0.182223	0.688245	−0.044283	0.045*	
H3B	0.394911	0.696219	−0.059962	0.045*	
C1	0.2601 (2)	0.53477 (8)	0.63385 (11)	0.0291 (4)	
H1A	0.265330	0.557750	0.698925	0.035*	
C2	0.2592 (2)	0.46329 (8)	0.63512 (11)	0.0294 (4)	
C3	0.2514 (2)	0.42535 (8)	0.53739 (12)	0.0292 (4)	
C4	0.2374 (2)	0.43679 (8)	0.34112 (12)	0.0321 (4)	
H4	0.235928	0.388404	0.335394	0.039*	
C5	0.2327 (3)	0.47623 (8)	0.25186 (12)	0.0354 (4)	
C6	0.2319 (2)	0.55061 (8)	0.25178 (11)	0.0270 (4)	
C7	0.2372 (2)	0.58073 (7)	0.35309 (11)	0.0274 (4)	
H7	0.235217	0.629125	0.358517	0.033*	
C8	0.24524 (19)	0.54122 (7)	0.44637 (11)	0.0256 (3)	
C9	0.2443 (2)	0.46788 (8)	0.44236 (11)	0.0273 (4)	
C10	0.2671 (2)	0.42813 (9)	0.74030 (12)	0.0351 (4)	
C11	0.2604 (2)	0.65153 (8)	0.55207 (12)	0.0326 (4)	
H11A	0.139091	0.669951	0.515682	0.039*	
H11B	0.375099	0.667878	0.514330	0.039*	
C12	0.2764 (3)	0.68081 (9)	0.66298 (13)	0.0422 (4)	
H12A	0.280299	0.730842	0.659726	0.063*	
H12B	0.397817	0.663933	0.699410	0.063*	
H12C	0.161388	0.666340	0.700562	0.063*	
C13	0.1897 (3)	0.56517 (9)	0.05373 (13)	0.0469 (5)	
H13A	0.050313	0.575445	0.031684	0.056*	
H13B	0.208003	0.514977	0.051602	0.056*	
C14	0.3288 (4)	0.59967 (10)	−0.02121 (14)	0.0534 (5)	
H14A	0.467697	0.587416	−0.001074	0.064*	
H14B	0.298153	0.582794	−0.092826	0.064*	
C15	0.2046 (3)	0.66574 (9)	0.16535 (13)	0.0490 (5)	
H15A	0.234172	0.682163	0.237302	0.059*	
H15B	0.064439	0.676689	0.145859	0.059*	
C16	0.3382 (3)	0.70272 (9)	0.09163 (13)	0.0454 (5)	
H16A	0.310319	0.752384	0.093207	0.055*	
H16B	0.478515	0.695757	0.115461	0.055*	
O4	0.62110 (18)	0.72318 (8)	−0.14696 (11)	0.0544 (4)	
O5	0.7972 (2)	0.68395 (8)	−0.01286 (11)	0.0628 (5)	
O6	0.9454 (2)	0.71910 (9)	−0.14916 (14)	0.0697 (5)	
N4	0.7890 (2)	0.70827 (7)	−0.10335 (11)	0.0383 (4)	

### 4-(3-Carboxy-1-ethyl-6-fluoro-4-oxo-1,4-dihydroquinolin-7-yl)piperazin-1-ium nitrate Atomic displacement parameters (Å^2^)

**Table:**

	*U*^11^	*U*^22^	*U*^33^	*U*^12^	*U*^13^	*U*^23^
F1	0.1802 (16)	0.0320 (6)	0.0297 (6)	0.0089 (7)	0.0057 (7)	−0.0077 (4)
O1	0.0642 (8)	0.0373 (7)	0.0399 (7)	0.0008 (6)	0.0013 (6)	0.0116 (5)
O2	0.0670 (9)	0.0511 (8)	0.0305 (7)	0.0034 (6)	0.0035 (5)	0.0060 (5)
O3	0.0526 (7)	0.0269 (6)	0.0409 (7)	−0.0004 (5)	0.0006 (5)	0.0042 (4)
N1	0.0280 (6)	0.0271 (7)	0.0252 (6)	0.0004 (4)	0.0003 (5)	−0.0005 (4)
N2	0.0462 (8)	0.0281 (7)	0.0244 (6)	−0.0018 (5)	0.0027 (5)	−0.0008 (5)
N3	0.0396 (7)	0.0417 (8)	0.0321 (7)	−0.0027 (6)	0.0042 (5)	0.0076 (5)
C1	0.0272 (7)	0.0348 (8)	0.0254 (7)	0.0004 (6)	0.0012 (5)	0.0012 (6)
C2	0.0246 (7)	0.0338 (8)	0.0296 (8)	0.0007 (5)	0.0011 (6)	0.0049 (6)
C3	0.0233 (7)	0.0284 (8)	0.0358 (8)	0.0004 (5)	0.0016 (6)	0.0039 (6)
C4	0.0370 (8)	0.0241 (7)	0.0352 (8)	0.0010 (6)	0.0020 (6)	−0.0005 (6)
C5	0.0495 (9)	0.0287 (8)	0.0281 (8)	0.0020 (7)	0.0036 (6)	−0.0061 (6)
C6	0.0251 (7)	0.0287 (8)	0.0272 (7)	−0.0010 (5)	0.0020 (5)	0.0011 (5)
C7	0.0296 (7)	0.0233 (7)	0.0291 (8)	−0.0009 (5)	0.0005 (5)	−0.0013 (5)
C8	0.0206 (6)	0.0287 (8)	0.0273 (8)	−0.0003 (5)	0.0010 (5)	−0.0004 (5)
C9	0.0232 (7)	0.0277 (8)	0.0311 (8)	0.0000 (5)	0.0011 (5)	0.0009 (5)
C10	0.0313 (8)	0.0395 (9)	0.0344 (9)	0.0010 (6)	0.0004 (6)	0.0091 (6)
C11	0.0389 (8)	0.0267 (8)	0.0320 (8)	0.0008 (6)	0.0004 (6)	−0.0012 (6)
C12	0.0558 (11)	0.0347 (9)	0.0357 (9)	0.0029 (7)	−0.0020 (7)	−0.0055 (6)
C13	0.0763 (13)	0.0368 (9)	0.0268 (8)	−0.0158 (8)	−0.0044 (8)	−0.0006 (6)
C14	0.0844 (15)	0.0448 (10)	0.0325 (9)	0.0064 (9)	0.0162 (9)	0.0002 (7)
C15	0.0866 (14)	0.0318 (9)	0.0296 (8)	0.0117 (8)	0.0129 (8)	0.0031 (6)
C16	0.0659 (12)	0.0318 (9)	0.0373 (9)	−0.0092 (8)	−0.0087 (8)	0.0048 (7)
O4	0.0380 (7)	0.0706 (9)	0.0537 (8)	−0.0043 (6)	−0.0058 (5)	0.0246 (6)
O5	0.0728 (10)	0.0773 (10)	0.0375 (7)	0.0144 (8)	−0.0061 (6)	0.0081 (6)
O6	0.0420 (8)	0.0876 (12)	0.0807 (11)	−0.0025 (7)	0.0152 (7)	0.0163 (9)
N4	0.0388 (8)	0.0353 (7)	0.0405 (8)	−0.0010 (6)	−0.0004 (6)	−0.0001 (6)

### 4-(3-Carboxy-1-ethyl-6-fluoro-4-oxo-1,4-dihydroquinolin-7-yl)piperazin-1-ium nitrate Geometric parameters (Å, º)

**Table:**

C5—F1	1.3506 (18)	C6—C7	1.4002 (19)
O1—H1	0.8200	C7—H7	0.9300
C10—O1	1.325 (2)	C7—C8	1.3972 (19)
C10—O2	1.208 (2)	C8—C9	1.406 (2)
O3—C3	1.2643 (19)	C11—H11A	0.9700
N1—C1	1.3371 (18)	C11—H11B	0.9700
N1—C8	1.4015 (18)	C11—C12	1.505 (2)
N1—C11	1.4895 (18)	C12—H12A	0.9600
N2—C6	1.3679 (18)	C12—H12B	0.9600
N2—C13	1.4587 (19)	C12—H12C	0.9600
N2—C15	1.457 (2)	C13—H13A	0.9700
N3—H3A	0.8900	C13—H13B	0.9700
N3—H3B	0.8900	C13—C14	1.508 (3)
N3—C14	1.482 (2)	C14—H14A	0.9700
N3—C16	1.485 (2)	C14—H14B	0.9700
C1—H1A	0.9300	C15—H15A	0.9700
C1—C2	1.370 (2)	C15—H15B	0.9700
C2—C3	1.429 (2)	C15—C16	1.497 (3)
C2—C10	1.486 (2)	C16—H16A	0.9700
C3—C9	1.448 (2)	C16—H16B	0.9700
C4—H4	0.9300	O4—N4	1.2465 (18)
C4—C5	1.354 (2)	O5—N4	1.2307 (19)
C4—C9	1.407 (2)	O6—N4	1.2311 (19)
C5—C6	1.425 (2)		
			
C10—O1—H1	109.5	O2—C10—O1	121.25 (14)
C1—N1—C8	119.29 (12)	O2—C10—C2	123.50 (15)
C1—N1—C11	121.19 (12)	N1—C11—H11A	108.5
C8—N1—C11	119.51 (11)	N1—C11—H11B	108.5
C6—N2—C13	125.43 (13)	N1—C11—C12	114.88 (12)
C6—N2—C15	121.32 (12)	H11A—C11—H11B	107.5
C15—N2—C13	109.78 (13)	C12—C11—H11A	108.5
H3A—N3—H3B	108.2	C12—C11—H11B	108.5
C14—N3—H3A	109.6	C11—C12—H12A	109.5
C14—N3—H3B	109.6	C11—C12—H12B	109.5
C14—N3—C16	110.09 (13)	C11—C12—H12C	109.5
C16—N3—H3A	109.6	H12A—C12—H12B	109.5
C16—N3—H3B	109.6	H12A—C12—H12C	109.5
N1—C1—H1A	117.6	H12B—C12—H12C	109.5
N1—C1—C2	124.81 (13)	N2—C13—H13A	109.6
C2—C1—H1A	117.6	N2—C13—H13B	109.6
C1—C2—C3	119.91 (13)	N2—C13—C14	110.09 (14)
C1—C2—C10	117.64 (13)	H13A—C13—H13B	108.2
C3—C2—C10	122.45 (14)	C14—C13—H13A	109.6
O3—C3—C2	122.81 (13)	C14—C13—H13B	109.6
O3—C3—C9	122.04 (14)	N3—C14—C13	110.89 (15)
C2—C3—C9	115.15 (13)	N3—C14—H14A	109.5
C5—C4—H4	119.5	N3—C14—H14B	109.5
C5—C4—C9	121.03 (14)	C13—C14—H14A	109.5
C9—C4—H4	119.5	C13—C14—H14B	109.5
F1—C5—C4	116.29 (14)	H14A—C14—H14B	108.0
F1—C5—C6	119.75 (13)	N2—C15—H15A	109.3
C4—C5—C6	123.96 (13)	N2—C15—H15B	109.3
N2—C6—C5	123.92 (13)	N2—C15—C16	111.55 (15)
N2—C6—C7	121.73 (13)	H15A—C15—H15B	108.0
C7—C6—C5	114.31 (12)	C16—C15—H15A	109.3
C6—C7—H7	118.6	C16—C15—H15B	109.3
C8—C7—C6	122.83 (13)	N3—C16—C15	111.25 (14)
C8—C7—H7	118.6	N3—C16—H16A	109.4
N1—C8—C9	118.62 (12)	N3—C16—H16B	109.4
C7—C8—N1	120.62 (13)	C15—C16—H16A	109.4
C7—C8—C9	120.76 (13)	C15—C16—H16B	109.4
C4—C9—C3	120.68 (14)	H16A—C16—H16B	108.0
C8—C9—C3	122.21 (13)	O5—N4—O4	119.25 (15)
C8—C9—C4	117.11 (13)	O5—N4—O6	120.21 (15)
O1—C10—C2	115.24 (14)	O6—N4—O4	120.52 (15)
			
F1—C5—C6—N2	−1.5 (2)	C5—C4—C9—C8	0.3 (2)
F1—C5—C6—C7	−179.23 (15)	C5—C6—C7—C8	0.8 (2)
O3—C3—C9—C4	−0.3 (2)	C6—N2—C13—C14	−141.27 (16)
O3—C3—C9—C8	−179.83 (13)	C6—N2—C15—C16	141.16 (15)
N1—C1—C2—C3	0.1 (2)	C6—C7—C8—N1	178.83 (12)
N1—C1—C2—C10	179.87 (13)	C6—C7—C8—C9	−1.3 (2)
N1—C8—C9—C3	0.18 (19)	C7—C8—C9—C3	−179.68 (13)
N1—C8—C9—C4	−179.42 (12)	C7—C8—C9—C4	0.7 (2)
N2—C6—C7—C8	−177.03 (13)	C8—N1—C1—C2	0.2 (2)
N2—C13—C14—N3	−58.8 (2)	C8—N1—C11—C12	−178.10 (13)
N2—C15—C16—N3	56.1 (2)	C9—C4—C5—F1	178.70 (15)
C1—N1—C8—C7	179.51 (12)	C9—C4—C5—C6	−0.8 (2)
C1—N1—C8—C9	−0.34 (18)	C10—C2—C3—O3	−0.1 (2)
C1—N1—C11—C12	0.7 (2)	C10—C2—C3—C9	179.98 (13)
C1—C2—C3—O3	179.71 (14)	C11—N1—C1—C2	−178.54 (13)
C1—C2—C3—C9	−0.23 (19)	C11—N1—C8—C7	−1.70 (19)
C1—C2—C10—O1	−177.93 (13)	C11—N1—C8—C9	178.45 (12)
C1—C2—C10—O2	2.2 (2)	C13—N2—C6—C5	14.7 (2)
C2—C3—C9—C4	179.68 (13)	C13—N2—C6—C7	−167.66 (16)
C2—C3—C9—C8	0.10 (19)	C13—N2—C15—C16	−58.8 (2)
C3—C2—C10—O1	1.9 (2)	C14—N3—C16—C15	−53.7 (2)
C3—C2—C10—O2	−178.01 (15)	C15—N2—C6—C5	171.54 (16)
C4—C5—C6—N2	178.02 (15)	C15—N2—C6—C7	−10.8 (2)
C4—C5—C6—C7	0.2 (2)	C15—N2—C13—C14	59.7 (2)
C5—C4—C9—C3	−179.32 (14)	C16—N3—C14—C13	55.2 (2)

### 4-(3-Carboxy-1-ethyl-6-fluoro-4-oxo-1,4-dihydroquinolin-7-yl)piperazin-1-ium nitrate Hydrogen-bond geometry (Å, º)

**Table:**

*D*—H···*A*	*D*—H	H···*A*	*D*···*A*	*D*—H···*A*
O1—H1···O3	0.82	1.80	2.5640 (17)	154
N3—H3*B*···O4	0.89	1.97	2.8526 (19)	169
N3—H3*A*···O6^i^	0.89	2.08	2.937 (2)	161
C13—H13*B*···F1	0.97	1.96	2.726 (2)	135
C15—H15*A*···O4^ii^	0.97	2.47	3.252 (2)	138

Symmetry codes: (i) *x*−1, *y*, *z*; (ii) *x*−1/2, −*y*+3/2, *z*+1/2.
